# A scoping review of the literature on the impact of the COVID-19 quarantine on the psychological wellbeing of medical students

**DOI:** 10.1186/s12909-022-03803-y

**Published:** 2022-11-09

**Authors:** Divya I. Vythilingam, Amog Prakash, Milad Nourianpour, William U. Atiomo

**Affiliations:** 1 Medical Student, School of Medicine, University of Nottingham, B Floor, Medical School, Queen’s Medical Centre, NG7 2UH Nottingham, UK; 2 Medical Student, College of Medicine, Mohammed Bin Rashid University of Medicine and Health Sciences, Dubai, United Arab Emirates; 3 College of Medicine, Mohammed Bin Rashid University of Medicine and Health Sciences, Dubai, United Arab Emirates

**Keywords:** COVID-19, Quarantine, Psychological, Wellbeing, Medical, Students, Impact, Pandemic

## Abstract

**Background:**

The goal of this study was to identify the nature and extent of the available published research on the impact of social isolation, on the psychological wellbeing of medical students, who had to quarantine due to the COVID-19 pandemic.

**Methods:**

Design. Scoping review. Search strategy. The PRISMA-ScR (Preferred Reporting Items for Systematic reviews and Meta-Analyses extension for Scoping Reviews), guideline, was used to structure this study. A search strategy was carried out across six bibliographic databases. PubMed, Embase, ERIC, Scopus, Cochrane Database of Systematic Reviews and Web of Science. The following search terms were used, “medical student*” AND “impact” AND “quarantine” AND “COVID-19”. Searches were initially confined to articles published (excluding conference abstracts) between 1 January 2019- 21 August 2021 but updated in September 2022 with the original search terms expanded to include “isolation” or “lockdown” as well as “quarantine” and the period of search extended to 21 August 2022. A search of secondary references was conducted. Data from the selected studies were extracted, and the following variables recorded; first author and year of publication, country of study, study design, sample size, participants, mode of analysing impact of quarantine from COVID-19 on mental health and results of the studies.

**Results:**

A total of 223 articles were identified in the original search in 2021 and 387 articles, in the updated search in 2022. Following the exclusion of duplicates and application of the agreed inclusion and exclusion criteria, 31 full-text articles were identified for the final review, most of which were cross sectional studies. Sample sizes ranged from 13 to 4193 students and most studies used a variety of self-administered questionnaires to measure psychological wellbeing. Overall, 26 of the 31 articles showed that quarantine had a negative impact on the psychological well-being of medical students. However, two studies showed no impact, and three studies showed an improvement.

**Conclusion:**

The evidence is growing. Quarantine because of the COVID-19 pandemic may have had a negative impact on the psychological wellbeing of medical students, but this is not certain. There is therefore a need for more studies to further evaluate this research question.

## Background

The COVID-19 pandemic as initiated by SARS-CoV-2, was first reported in Wuhan, Hubei Province, in China in December 2019. Since then, globally, nations have been combatting it from a health and economic standpoint [1]. Due to its highly contagious nature, many individuals were forced into quarantine, with the potential for increased social isolation and loneliness among citizens, which has been linked to worse cardiovascular and mental health outcomes [2]. Further, the SARS outbreak had a significant psychological impact on medical staff including the prevalence and rise of depression, acute stress disorder, alcohol dependency and post-quarantine mental distress [3].

In the context of medical schools, as strict measures were put into place, medical schools which relied heavily on face-to-face teaching had to alter their traditional approach of teaching and learning activities towards online platforms [4, 5]. As a result, rapid changes were implemented, resulting in dramatic educational and potential psychological disturbances for medical students, particularly those in quarantine [6, 7]. This was further aggravated by the substantial academic coursework, the need to maintain their academic performance, and the potential for significant emotional stress among medical students, all of which were made worse by the pandemic [8]. Further, coupled with the fear and uncertainty of the pandemic and its future course of action as depicted through a large media presence, this had the potential to take a greater toll on their mental and social wellbeing. Wellbeing for the purpose of this study is defined as a state of positive feelings and the ability for an individual to meet their full potential in the world [9]. A meta-analysis study by Moutinho illustrated that 34.6% of medical students suffered from depressive symptoms whilst 37.2% experienced anxiety symptoms prior to the SARS-CoV-2 outbreak, highlighting the significance of addressing and combatting the further negative impact of the pandemic on the mental health of these students [10].

However, the impact of social isolation necessitated by the quarantine required by medical students as a result of the COVID-19 pandemic is uncertain. Although it is tempting to assume an overall negative impact, some medical students may have been able to quarantine with family and friends which may have attenuated the negative impact of social isolation during the quarantine. Although there had been several individual primary studies addressing this topic, prior to starting our study, we were unable to find a systematic synthesis of the literature providing a comprehensive overview of the impact of quarantine on the psychological wellbeing of medical students during the COVID-19 pandemic. As medical students are our health care practitioners a negative psychological impact of quarantine during the COVID-19 pandemic is likely to result in anxiety and depression. Thus, if not addressed this creates long-term negative consequence on their overall quality of life and a long-term impact which may negatively affect the future quality of care given to the greater community. A scoping review of the literature was therefore conducted to identify the nature and extent of the available research evidence in this area. For this study, quarantine was defined as the period in which an individual is kept in isolation to prevent the spread of a contagious disease, however, the definition was inclusive of those students who are quarantining within their family groups.

## Methods

**Fig. 1:**
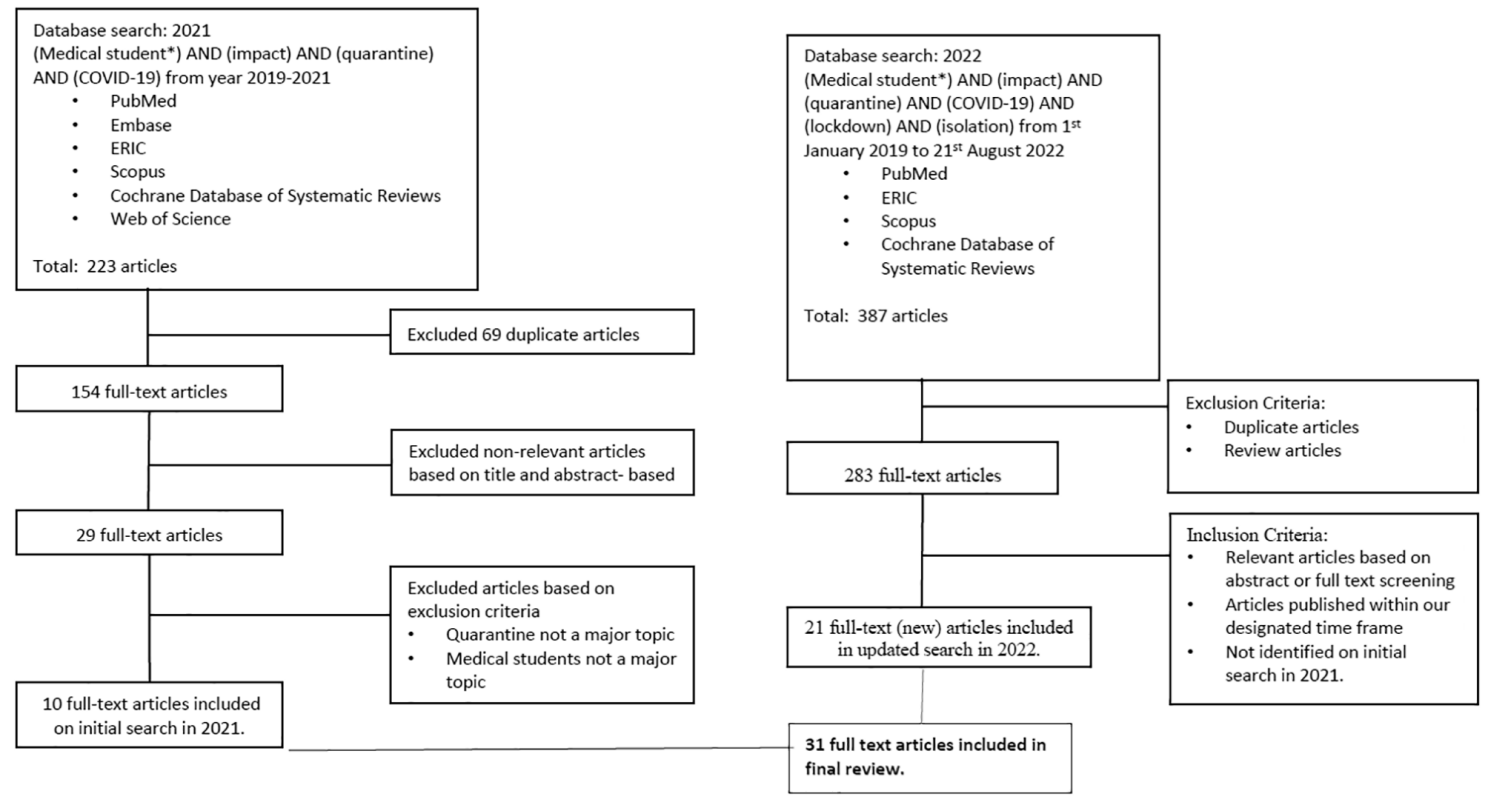
PRISMA flowchart

This study did not receive nor require ethics approval, as it did not involve humans & animals. The PRISMA-ScR (Preferred Reporting Items for Systematic reviews and Meta-Analyses extension for Scoping Reviews)[11] guideline, was used to structure this study. A scoping review was carried out to explore the extent of published data across literature to assess the impact of COVID-19 on the psychological wellbeing of medical students. The nature of this review was crucial as it allowed for a systematic analysis and summarisation of appropriate information across various publications through its methodological framework [12, 13]. Further, this methodology was also guided by the utilisation of the PRISMA-P 2015 checklist (Fig. 1), allowing for a structured and focused approach to this study, based on the outlined inclusion and exclusion criteria.

To ensure a viable and appropriate research process, a search strategy was developed, consisting of the search terms, “medical student*” AND “impact” AND “quarantine” AND “COVID-19”. Searches were confined to articles published (excluding conference abstracts) between 1 January 2019- 21 August 2021 on PubMed, Embase, ERIC, Scopus, Cochrane Database of Systematic Reviews and Web of Science. Further, for this study the participants were limited to undergraduate and postgraduate medical students whereby data gathered was confined to either through online questionnaires and/or phone calls. Following this, a search of secondary references was conducted and despite finding articles addressing the impact of COVID-19 on medical students, no further articles were found which discussed specifically the impact of quarantine due to COVID-19 on medical students. All searches were initially carried out by the first author (D.V.).

The search was however independently updated in September 2022 by two other authors (A.P. and M.N.) with the original search terms expanded to include “isolation” or “lockdown” as well as “quarantine” and the period of search was extended to articles published between 1 January 2019–21 August 2022 on PubMed, ERIC, Scopus and Cochrane Database of Systematic Reviews, to ensure we did not miss any important publications. All the final articles were then reviewed, and the following information was extracted from each and charted on a table initially on the Microsoft Excel software, which was then transferred to Microsoft Word; first author and year of publication, country of study, study design, sample size, participants and mode of analysing the impact of quarantine, isolation or lockdown, from COVID-19 on mental health or wellbeing (Table 1). Further, the sampling technique used for each study, alongside the use of a pilot group and a control group are also specified. Additionally, as each study analysed the impact of quarantine, isolation or lockdown, through various psychological scales and scores, these were also extracted from the studies and detailed alongside the descriptive findings, in addition to the various statistical tools and analytical methods utilised. Lastly, as data was analysed and collated, it was ensured that findings derived from each study were significant in nature as classified by their associated statistical tools, to ensure validity and reliability.

**Table 1:** Summary of characteristics of included articles

First author and year	Country of Study	Study design	Sample size; Participants.	Mode of analysing impact	Methodology
Alejandro-Salinas (2022) (20)	Peru.	Cross -sectional.	281; medical students.	PTSD evaluated with the Impact of Event Scale - Revised (IES-R) a 22-item self-administered questionnaire.	Data exported to a Microsoft Excel spreadsheet from data obtained from Google Forms, then a statistical package STATA v15.0 (StataCorp, TX, USA) used for analysis.
Ali (2020) (21)	Pakistan.	Cross-sectional.	182; First, second- and third-year medical students.	Depression, Anxiety and Stress Scale (DASS).	Self-administered electronic questionnaires. Sample size calculated through non- probability consecutive sampling (50% of selected population). Frequency and percentages from the DASS were calculated to correlate the effects on daily routine. Chi square utilised to calculate association across different year groups. Pilot study conducted. No use of a control group.
Arima (2020) (8)	Japan.	Cross-sectional.	571; All medical students.	K-6 scale for psychological distress. Rosenberg Self Esteem Scale (RSES). General Self Efficacy Scale (GSES).	Self-administered electronic questionnaires. No sample size calculation. Demographics and variables were utilised to assess the determinant factor for psychological distress through logistic regression. Regression analysis utilised to evaluate RSES and GSES scores. Validity and reliability pertaining to measures of distress were assessed over four weeks prior to survey administration. No use of a control group.
Banerjee (2021) [22]	Mauritius.	Cross -sectional.	663; medical students from 1st to 10th semester.	Questionnaire designed following a literature review measuring guilt, depression, time management, focus, sleep, comprehension, fear and motivation.	Eight items on questionnaire were used to assess the psychological impact on medical students due to COVID-19. The questionnaire was validated by five subject experts and initially tested via the use of a pilot study with 10 students. The reliability of the questionnaire was ascertained by Cronbach’s alpha.
Bolatov (2020) [18]	Kazakhstan.	Cross-sectional.	798 (intervention group). 619 (control group). First to fifth year medical students.	The Patient Health Questionnaire-9 (PHQ-9) scale. The Generalized Anxiety Disorder, the 7-item (GAD-7) Scale. The Patient Health Questionnaire-15 (PHQ-15) scale. Fear of COVID-19 was assessed using a 5-point adapted Snell’s questionnaire. The Copenhagen Burnout Inventory (CBI-S).	Self-administered electronic questionnaires. No sample size calculation. Burnout syndrome, depression, anxiety, somatic symptoms, and satisfaction with academic performance were analysed. The chi-squared test or independent sample t test was utilised to evaluate the differences between variables. In comparing results within one sample, ANOVA analysis and the Bonferroni post hoc test was utilised. In assessing independent variable associations, a logistic regression analysis was carried out. Control group included.
Dworkin (2021) [16]	13 Countries.	Participants were asked to take 3–4 photographs over a two-week period depicting daily life during the pandemic, and then write brief reflections about each photograph.	26; Medical students.	A directed content analysis approach.	Qualitative study of photographic reflection taken.
Ferreira (2021) [23]	Brazil.	Cross-sectional.	216; medical students from 9th to 12th semester.	Psychiatric diagnosis, use of psychotropic drugs, and legal or illegal psychoactive substances.	Questionnaires consisted of closed-answer questions (multiple-choice, single-answer, dichotomous-answer), matrix (Likert scale), and open-answer questions.
Frajerman ( 2022) [24]	France.	Online national cross-sectional study	2623; Medical students.	Used Kessler’s K6, a validated scale in 6 items to evaluate psychological distress.	Statistical significance was tested in bivariate analyses using the chi-square test. Logistic regression models were performed for statistically significant associations in bivariate analyses for the K6 scale.
Kalok (2020) [6]	Malaysia.	Cross-sectional.	627; Medical clinical year students.	Depression, Anxiety and Stress Scale-21 (DASS 21). Short Warwick Edinburgh Mental Well-Being Scale (SWEMWBS). Perception of the Effect of MCO* on Self-Wellbeing. Source of Social Support. *MCO: Movement Control Order- Government instigated partial lockdown.	Self-administered electronic questionnaires. Sample size was calculated based on precision, confidence interval and dropout figures. Questionnaire Validation of Social Support and Perception of MCO on Self-Wellbeing performed. DASS 21, SWEMBS, and MCO effects were analysed for normality using the Kolmogorov–Smirnov test. Internal consistency for the effect of the MCO was evaluated using a reliability test. Correlations between depression and mental wellbeing, anxiety, stress, and the effects of the MCO were analysed using Pearson’s correlation whilst significant associations were highlighted through univariate analysis where statistically significant variables were utilised through multivariate logistic regression analyses. Student’s t-tests and one-way ANOVA was utilised to analyse the variability between groups for demographic variables and social support. No use of a control group.
Korobchansky (2021) [25]	Ukraine.	Cross-sectional.	273; Medical students.	Examining factors including students’ lifestyle, day regime, features of organising distance learning and changes in physical and psycho-emotional state.	Self-administered electronic questionnaires. No sample size calculation. Assessing impact of adverse factors on health status and lifestyle of students through examining factors including students’ lifestyle, day regime, features of organising distance learning and changes in physical and psycho-emotional state. No use of a control group.
Kosendiak (2021) [26]	Poland.	Cross-sectional study.	2920; Medical students.	Coping Orientation to Problems Experienced questionnaire (Mini-COPE), WHO’s Alcohol Use Disorders Identification Test, Fagerström Test for Nicotine Dependence.	Chi-square test was used with Bonferroni correction. To test statistically significant differences between groups the non-parametric Skillings–Mack test with the post-hoc Dunn’s test (for variables not meeting the conditions of normal distribution) was used. Spearman’s rank order test was used for correlation analysis.
Kosendiak (2022) [27]	Poland.	Cross-sectional study.	225; Medical students in second year	Response to questions on alcohol intake, smoking and sleeping duration.	Chi-square test of independence was performed to assess statistically significant differences between expected and observed values contained in a contingency table.
Leroy (2021) [28]	France.	Cross-sectional study.	4193; Medical students.	The mental health outcomes evaluated were suicidal thoughts, severe self-reported distress (as assessed by the Impact of Events Scale-Revised), stress (Perceived Stress Scale), anxiety (State-Trait Anxiety Inventory, State subscale), and depression (Beck Depression Inventory).	Multivariable logistic regression analyses were performed to test the association between the type of university studies (healthcare studies: medical and non-medical, and non-healthcare studies) and poor mental health outcomes.
Meo (2020) [7]	Saudi Arabia.	Cross-sectional.	530; First to fifth year medical students.	Psychological wellbeing, stress- allied queries and learning behaviour were analysed through Five Point Likert Scale.	Self-administered electronic questionnaires. Sample size obtained using simple random sampling and calculated using a power formula. Pilot study conducted. No use of a control group.
Miskulin (2020) [29]	Brazil.	Cross-sectional.	347; First to sixth year medical students.	Hospital Anxiety and Depression Scale (HADS).	Self-administered electronic questionnaires. No sample size calculation. Chi-square test and Mann-Whitney test were used to categorise and make comparisons of continuous variables respectively. Correlations were derived using Spearman correlation test. To evaluate the influence of year class and location of students during quarantine, binary logistic regression was utilised. No use of a control group.
Peng (2020) [30]	China.	Cross-sectional study.	430; Medical students (442 non-medical students.	Questions on attitude toward COVID-19 e.g. “Do you hope the outbreak to stop quickly so you can return to school soon” and “Do you think you will be more capable to endure such public health emergence?“	Questionnaire study.
Pereira (2020) [19]	Brazil.	Cross-sectional.	860; First to fourth year medical students.	Prevalence of CMDs* analysed through Self-Reporting Questionnaire (SQR-20) designed by WHO** to screen for emotional distress. *CMDs: Common mental disorders**WHO: World Health Organisation	Self-administered electronic questionnaires. No sample size calculation. Groups were analysed through utilising the Chi-squared test and Kruskall-Wallis test for categorical variables and continuous variables respectively. Control group included.
Pravinraj (2022) [31]	India.	Cross-sectional.	204; Medical students (Prefinal and final year).	Depression Anxiety Stress Scale (DASS 21).	Data was collected in preformed, self-administered, pretested questionnaires and Spearman`s correlation, Ordinal logistics regression was applied to find the predictors of Depression, Anxiety and Stress.
Qanash (2020) [4]	Saudi Arabia.	Cross-sectional.	362; First to sixth year medical students.	Four-Item Patient Health Questionnaire for Anxiety and Depression (PHQ-4).	Self-administered electronic questionnaires. Sample size was calculated using non- probability convenient sampling. Two Sample t-test used for continuous variables that had a normal distribution. Welch Two Sample t-test used when both groups had unequal variance. Wilcoxon rank sum test was utilised for continuous variables that were not normally distributed. Chi-square or Fisher’s exact test were utilised to analyse categorical variables. Kruskal-Wallis test was utilised for ordinal attributes. Excluded students with personal history of psychological illness. Pilot study was conducted. No use of a control group.
Rolland (2022) [32]	France.	Cross-sectional study.	1712; Medical students (also recruited non-medical students.	Kessler’s K6 scale	Descriptive information was provided as percentages. Statistical significance was tested in bivariate analyses using the Chi2 test and Fisher’s exact test to compare between groups’ prevalence. Subsequently, logistic regression models adjusted for age and sex were performed for statistically significant associations in bivariate analyses.
Ross (2021) [33]	South Africa.	Cross-sectional study.	256; 5th year medical students	Question about being in a good ‘headspace’ to engage in online learning.	Data were downloaded from Google forms onto an Excel spreadsheet, cleaned and analysed descriptively by using Statistical Package for Social Sciences (SPSS) version 27 to determine central tendency, variation and associations and calculate odds ratios.
Sam (2022) [14]	Malaysia.	Qualitative study.	13; medical students.	The recorded interview data were thematically analysed using the six phases of Braun and Clarke’s Thematic Analysis.	In-depth individual interview via Microsoft Teams (Microsoft Corp.) with semi-structured questions.
Šimić (2021) [34]	Bosnia and Herzegovina.	Cross-sectional study.	246; medical students.	The impact scale of the traumatic event (IES - Impact of Event Scale). Furthermore, the questionnaire contained six questions on the negative impact of a pandemic on mental health.	Participants completed a modified anonymous online questionnaire.
Soltan (2021) [35]	Egypt.	Cross -sectional.	282; medical students.	Psychometric tools for the assessment of depression, anxiety and stress (Depression Anxiety Stress Scales DASS-21) and the Impact of Event Stress Scale-Revised (IES-R)	The variables were expressed in number, percentage, mean and standard deviation. Association between qualitative variables was assessed using Chi-square test. Fisher’s exact test was used in the case that any of the expected cells were less than five. A logistic regression was performed to ascertain the effects of possible risk factors on depression, anxiety, and other outcomes.
Tahir (2022) [36]	Pakistan.	Cross-sectional.	1100; Medical students from 5 medical schools.	The influence of COVID-19 Pandemic on sleep, physical activity, and nutrition, substance abuse, dealing with finances, spirituality and family life using Likert scales.	Convenience sample. Self-administered online questionnaires.
Thind (2021) [37]	Saint Kitts and Nevis.	Cross-sectional.	104; Medical students (2nd, 3rd and 4th years).	2 questions on the survey; (1) How anxious did you feel during the lockdown? (2) How depressed did you feel during the lockdown?	Survey distributed using co-students, friends’ circle, and through social media platforms.
Wang (2021) [38]	China.	Cross-sectional.	403; Medical students.	Perceived Stress Scale (PSS-10).	The sample size was computed by conducting linear multiple regression prior to power analysis using the G* Power 3.1 software. The potential stressors in this study were adapted from the Source of Stress Questionnaire.
Wurth (2021) [15]	Switzerland.	Mixed methods.	803; Medical students (2nd to 6th years).	Perceived Stress Scale (PSS).	A survey containing on one hand open-ended questions, yielding qualitative data, and on the other hand, Likert type items and Yes-No responses to closed questions
Xiao (2020) [39]	China.	Cross-sectional.	933; Medical students.	Patient Generalized Anxiety Disorder-7 and Health Questionnaire-9.	Multivariable logistic regression analyses were performed to test the association between the type of university studies (healthcare studies: medical and non-medical, and non-healthcare studies) and poor mental health outcomes.
Zhao (2021) [40]	China.	Cross-sectional.	666; First to third year medical students.	Depression measured through Patient Health Questionnaire-9 (PHQ-9). Simplified Coping Style Questionnaire utilising a Likert scale. Ego Resilience 89 Scale.	Self-administered electronic questionnaires. Sample size was calculated using stratified sampling. Assessing prevalence of depression and exploring the role of coping styles as facilitators between resilience and depression. Comparison among groups was analysed through two-tailed t-tests and one-way analysis of variance tests. Hierarchical linear regression was utilised to assess the mediating role of coping styles alongside resilience and depression. Structural equation modelling was utilised to depict the role that coping styles had in the relationship between resilience and depression. Validity and reliability of questionnaire was analysed.
Žuljević (2021) [17]	Croatia.	Cross-sectional pre and post survey.	437 in pre survey and 235 after; Medical students.	Oldenburg Burnout Inventory and Copenhagen Burnout Inventory.	Data were collected before lockdown in December 2019 and January 2020 and again after the end of lockdown in June 2020. Study was initially planned in 2019 with the aim of comparing medical student study satisfaction and burnout between clinical and preclinical study years, as well as using a follow-up survey to assess possible changes as the academic year goes on.

## Results

A total of 223 articles were identified across the six databases following our initial search in 2021, from which 69 duplicates were excluded resulting in 154 full-text articles. These articles were then reviewed based on their titles and abstracts in accordance with the exclusion criteria, resulting in 29 full-text articles. The full text of all 29 articles were then reviewed and screened again alongside the exclusion criteria, excluding those which did not include quarantine and medical students as major topics. Overall, 10 full-text articles were analysed on our initial search as displayed through the PRISMA process in Fig. 1.

However, the independent and updated search in 2022, identified 387 articles and following the exclusion of duplicates and review articles, 283 full text articles remained. On screening of the abstracts or full texts of these articles for relevance and publication within the new designated time frame, 21 additional full text articles were identified. Overall, 31 full-text articles were therefore analysed in this study as displayed through the PRISMA process in Fig. 1. Sample sizes ranged from 13 to 4193 students and most studies used a variety of self-administered questionnaires to measure psychological wellbeing including depression, anxiety, stress, insomnia, and emotional stability.

Most (27/31) of the studies identified in our final list of included studies were primarily cross-sectional studies surveys without a control group. There was however one qualitative study [14], one mixed methods study [15], one qualitative study of photographic reflections taken during the pandemic[16] and one study that involved a pre and post pandemic survey (therefore a “control group”) [17]. In the last study [17], data had been initially collected before lockdown in December 2019 and January 2020 and again after the end of lockdown in June 2020. The study was initially planned in 2019 with the aim of comparing medical student study satisfaction and burnout between clinical and preclinical study years, as well as using a follow-up survey to assess possible changes as the academic year goes on. The other studies that had a “control group” included, a study[18] where data on burnout, depression, anxiety was collected via questionnaires on 1st to 5th year medical students during a period of traditional learning (October–November 2019) before the pandemic and compared with a second study completed during an online learning period during the COVID 19 pandemic (April 13–19, 2020) and a study[19] which had started gathering prospective data on the prevalence of common mental disorders in medical students three years before and during COVID-19 quarantine.

Following a detailed analysis of the 31 included articles, we found that the majority (26/31) of articles highlighted a negative impact of quarantine, isolation, or lockdown on the psychological wellbeing of medical students as seen through various self-administrated online tools and measurement scales (Table 1). However, the three studies [17–19] we identified with “control data” showed either an improvement [18] or no difference on the psychological wellbeing of medical students with quarantine, isolation, or lockdown because of the COVID-19 pandemic.

The prevalence of depression among medical students was analysed and found to be significant across several studies; for example, Arima highlighted that 28.5% of students experienced a significant degree of psychological distress, Kalok found that greater than 50% of medical students displayed symptoms of psychological distress whilst Korobchansky denoted a prevalence of depression in 26.7%; 23.6% through Meo’s analysis and evident in 9.6% of medical students through Zhao’s study [6–8, 21, 25, 40]. However, Arima also found that following logistic regression analysis, higher scores on the Rosenberg Self Esteem and General Self Efficacy Scales were independent factors that correlated with lower levels of psychological distress. Likewise, through regression analysis, Zhao noted that coping styles (p < 0.001) and levels of resilience (p = 0.04) were both independent predictors of depression [40]. Further through a structural equation modelling analysis, Zhao found that the significance of resilience on depression was moderately mediated by coping styles (p = 0.007) [40].

Despite Ali’s study noting no significance in depression scores across students, a significance in the prevalence of stress (p = 0.001) and anxiety (p = 0.008) across students of different educational year groups was highlighted [21]. Further, measurement tools analysing both anxiety and depression denoted that 36% of medical students had scores greater than the HADS-D > 8 cut-off value and 23.6% were classified in the moderate to severe category in the PHQ-4 scale as highlighted by Qanash and Miskulin, respectively [4, 29]. Meo also depicted that 44.2% of students felt emotionally detached from their family, 38.1% experienced hopelessness, exhaustion or were emotionally unresponsiveness and 56.2% noted a deterioration in their work performance and studying subject contents [7].

Further, the presence of deterioration of vision, headaches, absent- mindedness, anxiety and sleep disturbance was studied alongside depression through Korobchansky’s analysis [25]. Similarly, other factors such as presence of hopelessness, exhaustion, emotional instability, anxiousness, and insomnia were highlighted through Meo’s study [7]. Arima explored the effect of self-esteem and self-efficacy as influential factors in predicting psychological distress among medical students [8]. Other factors that were to be beneficial in combatting the negative impact of quarantine including the availability and extent of social support either in the form of familial or governmental support [6]. As such, these factors were associated with a lower prevalence of depression and stress as well as a greater psychological wellbeing [6]. Further, interestingly it was noted that several studies, depicted that junior year medical students experienced greater levels of stress and anxiety when compared to their senior peers. Miskulin’s study in particular noting that 45.6% first-year medical students had a HADS-D > 8 when compared to the 32% prevalence as found across older year medical students (p = 0.015) [4, 6, 21, 29].

Contrastingly, five studies found either no impact of quarantine, lockdown or isolation during the COVID-19 pandemic, on the psychological wellbeing of medical students or an improvement. Interestingly, three of these studies were the only studies identified in our scoping review, with “control data”. Pereira’s longitudinal study illustrated a lack of significant variations in the prevalence of common mental disorders between 2018 (62.2%), 2019 (60.9%) and 2020 (59.2%) for the SQR-20 ≥ 7 cut off value (p = 0.762) [19]. Further, Bolatov’s study unpredictably depicted that quarantine had a significant positive impact whereby both depression (27.6%) and anxiety (15.5%) rates decreased during quarantine when compared to the prevalence prior, depression (49.3%) and anxiety (42.3%) [18]. Additionally, whilst this study revealed that the prevalence of the burnout syndrome and somatic symptoms decreased after transitioning from face-to-face learning to online learning, the prevalence of colleague associated burnout increased, highlighting the negative impact that quarantine and online learning had on interpersonal relationships and students’ communication [18]. Finally, the study by Žuljević, found no evidence for an increase in the level of burnout before and after lockdown, both in independent and paired samples [17].

However, despite these outlier studies, overall, a substantial negative impact on the psychological wellbeing of medical students appears to have resulted due to quarantine during the COVID-19 pandemic, particularly amongst younger students and those in the early years of their medical degree [4, 6, 8, 21, 29].

## Discussion

The evidence of the negative impact of quarantine on the psychological wellbeing of medical students is growing but still uncertain. Although majority of the articles in our scoping review identified a negative impact of quarantine, social isolation or lockdown on the psychological wellbeing of medical students, five of the 31 articles reviewed showed varying results. Two studies showed no difference in psychological wellbeing and three showed, a positive impact of quarantine on the mental health of medical students, which highlights the need for future comprehensive studies to further evaluate this research question.

A wide array of measurement tools and scales were utilized, to measure psychological wellbeing, highlighting the impact of social isolation, quarantine or lockdown during the COVID-19 pandemic, on a range of psychological symptoms including depression, anxiety, stress, insomnia, and emotional stability (Table 1). As such, these findings are significant as this study represents the first scoping review that focusses on the impact of quarantine on medical students amidst the COVID-19 pandemic and thus sheds light into this field from which further avenues can be explored. Additionally, the negative psychological impact that quarantine has probably had on these students are consequential, because as future health care practitioners, anxiety and depression can result in long-term effects which impact the quality of care given to the greater community.

With respect to previous research, no previous scoping reviews of the literature on this topic were found prior to the onset of our study and our understanding of the wider literature, on the impact of quarantine on medical students is currently limited to the findings of this study. There is therefore a need for future research in this area. Our findings must also be interpreted in the context of previous research on the psychological wellbeing in other disciplines. For example, a study conducted across students at a Spanish University concluded that undergraduate students had significantly higher depression, anxiety and stress scores when compared to Master’s students [41]. Interestingly, this study also noted that Arts and Humanities students had the greatest anxiety and depressive scores when compared to their peers across other facilities [41]. Further, another study conducted among French university students highlighted a high occurrence of severe self-reported depression, anxiety, stress, and distress as well as self-reported suicidal thoughts among those who were quarantined [42]. These studies and the finding from our scoping review that the three studies that found either no impact of quarantine, lockdown, or isolation during the COVID-19 pandemic, on the psychological wellbeing of medical students were the only studies with control data, make a strong case for future research.

The negative impact that quarantine has probably had on the psychological wellbeing of medical students could lead to long term health consequences as doctors, as depicted through studies that found an inversely proportional relationship between self-efficacy and depression related symptoms and how this can lead to the development of other negative psychological attributes [43]. Thus, highlighting how this negative impact might lead to less resilience among medical students which consequentially results in challenging situations arising when faced with unfavourable circumstances. In contrast, Edwards’s study depicted that those medical students who possessed high self- efficacy and self-esteem were more capable of handling various stresses, acquire better communication skills and interpersonal relationships which leads to an improvement in the physician- patient relationship [44].

It is interesting to note that a study conducted in Australia during the equine influenza outbreak depicted that individuals who were quarantined experienced greater levels of psychological distress compared to those who weren’t, emphasising the effect the associated isolation had as opposed to that caused by the outbreak alone [45]. Thus, in our analysis, despite the three studies that strayed from the findings of the majority, our study has shown that quarantine during the COVID-19 pandemic, has probably had a significant detrimental impact on the psychological wellbeing of medical students globally. Therefore, it is vital to address these psychological issues to prevent further damaging consequences to both medical students and the community they serve. To address and tackle these negative psychological impacts, the implementation of wellbeing programs among medical students, effective contact tracing and mental health assessments should be considered. For example, some wellbeing programs can include the incorporation of weekly wellbeing drop-in sessions as well as counselling services offered to students via self-referrals. Contact tracing measures might include contacting international students regularly to check on their wellbeing, whilst mental health assessments could include ensuring a system is in place to allow for prompt referrals of students who have mental health enquires. Further, another aspect is to focus on developing strategies to ensure medical students remain engaged, for instance through the implementation of interactive case discussions, and an increased engagement with pastoral support staff via online webinars.

### Strengths and limitations

The strengths of this study are associated with this study being the first scoping review which encompassed findings from a range of universities globally. This is vital as it allows for a greater understanding of the various consequences of quarantine, lockdown, or isolation during the COVID-19 pandemic, on the psychological wellbeing of medical students. As such, this is beneficial and significant for future studies which can aim to focus on developing methods to effectively eliminate and combat against negative impacts on the mental health of students. As well as this, this study also highlights the significance of developing appropriate strategies to enhance the positive elements that can be implemented into medical education, for instance through the incorporation of targeted online platforms and tutoring within universities. Finally, independently updating our search, in September 2022 by two other co-authors, with the original search terms expanded and the period of search extended, minimised the risk of our missing any important publications.

There were, however, some limitations of this scoping review. Firstly, many of the studies analysed in this review were cross-sectional in nature and thus this potentially could have led to casual associations. Future studies should therefore focus on being conducted in a longitudinal series, comparing the long-term psychological outcomes of students who experienced quarantine, lockdown, or isolation during the COVID-19 pandemic with students who did not (e.g., students who had graduated before the pandemic or new medical students starting medical school after the pandemic). Secondly, because online questionnaires were used, different modes for analysing data were present and thus student responses were mostly subjective. Further studies should ideally include interviews from students to allow for a greater understanding and interpretation of the factors affecting mental health and wellbeing. It was also difficult to accurately compare results across studies in a meta-analysis. Many of the studies also did not include a control group of medical students, pre-COVID-19 quarantine, highlighting the need for future studies to include two separate groups to allow for a contrast between the two, and a more accurate evaluation, albeit challenging to implement. It was also difficult to establish from the studies whether the effect been reported by the students was specifically due to a period of quarantine / isolation or the more general enforced restrictions in daily life due to a global pandemic.

## Conclusion

Our scoping review found growing literature on the impact of quarantine because of quarantine, lockdown, or isolation during the COVID-19 pandemic, on the psychological wellbeing of medical students. Most of the 31 studies included the final review were cross sectional, used a variety of measurement tools and did not include any controls. Overall, most studies (83%) found that the quarantine period during the COVID-19 pandemic probably had a negative psychological impact on the mental wellbeing of medical students. However, five of the 31 articles reviewed showed varying results, with two showing no difference in psychological wellbeing and three, a positive impact of quarantine on the mental health of medical students. This highlights the need for future comprehensive studies to further evaluate this research question. This is important to decrease and prevent the occurrence of psychological disorders among medical students. Therefore, in the meantime, it is recommended that medical schools implement targeted strategies and programs that aim to prevent and decrease psychological disorders among their students that may have arisen because of quarantine during the COVID-19 pandemic. By doing this, the potential long-term negative consequences on their overall quality of life may be reduced and the future quality of healthcare provided to the greater community, by these medical students, would be of safe and excellent standards as expected of medical students globally.

## Data Availability

All data generated or analysed during this study are included in this published article.
